# A Qualitative Study of a Mindfulness-Based Intervention in Educational Contexts in Chile: An Approach Based on Adolescents’ Voices

**DOI:** 10.3390/ijerph17186927

**Published:** 2020-09-22

**Authors:** Álvaro I. Langer, Sebastián Medeiros, Nelson Valdés-Sánchez, Rodrigo Brito, Christoph Steinebach, Cristian Cid-Parra, Antonella Magni, Mariane Krause

**Affiliations:** 1 Mind-Body Lab, Instituto de Estudios Psicológicos, Facultad de Medicina, Universidad Austral de Chile, Valdivia 5090000, Chile; 2 ANID, Millennium Science Initiative Program, Millennium Nucleus to Improve the Mental Health of Adolescents and Youths (Imhay), Santiago 8380455, Chile; 3 ANID, Millennium Science Initiative Program, Millennium Institute for Research in Depression and Personality (MIDAP), Santiago 7820244, Chile; sebastianmedeiros@gmail.com (S.M.); nlvaldes@uc.cl (N.V.-S.); mariane.krause@gmail.com (M.K.); 4 Center for Interdisciplinary Studies on the Nervous System (CISNe), Universidad Austral de Chile, Valdivia 5090000, Chile; 5 Psychiatry Department, Pontificia Universidad Católica de Chile, Santiago 8320000, Chile; 6 Carrera de Psicología, Universidad Santo Tomás, Santiago 8370003, Chile; 7 Centre for Research in Human Flourishing, University of Nottingham, Nottingham NG8 1BB, UK; rodrigobritopastrana@gmail.com; 8 Escuela de Psicología, Universidad Mayor, Santiago 7550000, Chile; 9 School of Applied Psychology, ZHAW Zürich University of Applied Sciences, 8037 Zürich, Switzerland; christoph.steinebach@zhaw.ch; 10 Corporación la Esperanza, Santiago 7550000, Chile; cristiancidparra@gmail.com; 11 Corporación Formando Chile, Santiago 8420200, Chile; agmagni1@gmail.com; 12 Escuela de Psicología, Pontificia Universidad Católica de Chile, Santiago 7820244, Chile

**Keywords:** mindfulness-based intervention, adolescence, education, prevention, mental health

## Abstract

The application of mindfulness-based interventions in school settings has increased considerably in recent years, showing that differences between the characteristics of programmes can impact on the receptivity and effectiveness of mindfulness training. However, few studies have explored the learning process from the perspective of the children and adolescents who participate in mindfulness practice. The goal of this paper is to analyse the subjective experience of a group of adolescents following the completion of a mindfulness-based intervention developed for schools in Chile. The intervention studied is the “.b curriculum”, which is part of the Mindfulness in School Project (MiSP) developed in the UK. Twenty adolescents participated in semi-structured interviews within their school, in which three key areas were explored: pedagogy, perceived effects, and mechanisms of action, each of them being analysed from the perspective of thematic analysis. The results support the view that pedagogy is a very relevant consideration in the implementation, development, and efficacy of mindfulness-based interventions within the school context. We propose that the inclusion of structure, contents, process/mindful practices, and teachers’ expertise provides the pedagogical-relational framework required for students to successfully develop mindfulness skills, which enables them to experience their cognitive, emotional, and somatic effects. These effects are linked to self-regulation strategies, based on paying attention to one’s somatic experience with kindness and curiosity, which works as an attentional anchor. It is hoped that these results will contribute to the spread of mindfulness research in adolescents in Latin America, thus facilitating cross-cultural and international comparisons.

## 1. Introduction

Childhood and adolescence are crucial in mental health development and may be determinant for mental problems in adulthood [1,2]. The World Health Organization (WHO) has declared that 10–20% of children and adolescents have been diagnosed with a mental disorder [3], while some authors suggest that the figure is even higher [4]. Thus, the implementation of strategies based on promotion of well-being and prevention of mental disorders in adolescents must be a worldwide priority. Given the formative role of the educational system in adolescents’ development, it became a necessity for schools to enhance the development of socio-emotional and behavioural skills as an essential part of the 21st century curriculum, which was achieved through programs commonly known as Social Emotional Learning (SEL) [5]. In this regard, one of the most promising strategies, systematically researched and tested in several countries, are mindfulness-based interventions (MBIs) [6]. Indeed, MBIs may lay the groundwork for self and social resilience by targeting specific psychological needs in connection with socio-emotional development during adolescence [7]. 

The term “mindfulness”, used to refer to a complex phenomenon, derives from the Buddhist tradition, specifically from the Pali word “sati”, which means “remembrance, memory, reminiscence, recollection, thinking of or upon (any person or thing) […]” [8]; in brief, reminding oneself to return one’s attention to the present moment from one’s habitual state of distraction. In the last four decades, this term has been defined from a secular/scientific perspective, on an operational level, as “paying attention in a particular way: on purpose, in the present moment and non-judgmentally” [9], which means that all experiences, including thoughts, feelings, and sensations, are acknowledged and accepted, minimising the mental interference of our usually negative inner comments [10]. Brown and Ryan [11] define it as a state of consciousness characterized by being open and receptive to the present experience. Mindfulness can be understood both as a trait and as a state that can be cultivated through practice. Typically, it is through MBIs, which are structured courses (usually composed of weekly group sessions) where contemplative skills are taught and discussed [9].

The benefits of mindfulness practice amongst adults have been widely reported, both in clinical and non-clinical contexts [12,13]. Recently, studies on the benefits of MBIs for children and adolescents have found positive results in various functional and developmental areas [14,15,16,17], particularly at the emotional self-regulatory level [14].

Black [18] claims that the repeated practice of centering one’s attention strengthens neurocognitive capacity, especially within executive functions that have an impact on diverse areas of cognitive development associated with improved academic achievement and school performance [19,20,21,22]. For instance, Lawlor [23] emphasises the role of mindfulness in the development of competences that are central to social and emotional aspects of learning, such as self-management, self-awareness, and decision-making, amongst others. In the emotional domain, Broderick and Frank [24] claim that mindfulness allows adolescents to develop the regulatory and emotional abilities that they require to successfully manage the challenges that characterise this developmental stage. These benefits include an increase in self-esteem, emotional competency, and self-realisation [25,26,27]. Likewise, mindfulness could strengthen tolerance to discomfort, as practice leads adolescents to pay attention to all their experiences, including those which they consider to be unpleasant [28,29,30]. Moreover, Black et al. [31] suggest that mindfulness not only promotes healthy behaviour, but may also prevent young people from developing habits that are harmful to their health, such as tobacco consumption.

Although mindfulness-based interventions aimed at children and adolescents have achieved encouraging results [15,32], most research has focused on their quantitative impact. Furthermore, few studies have explored the mechanisms of action involved in the subjective experience of the young people participating in these interventions. Also, the frequent use of standardized scales to measure the effectiveness of various mindfulness programs, which are usually over-standardized themselves, can result in an over-simplified understanding of the nature, implementation, and potential of these practices, thus neglecting a deeper comprehension and a more holistic approach [33,34,35]. In this sense, qualitative research can contribute to a deeper and more detailed understanding of the implementation of mindfulness within schools, as it can generate precise knowledge about the characteristics and needs of this age group [36,37,38]. Thus, it may be possible to complement and support effectiveness research that has been carried out through randomised control trials; for example, by incorporating qualitative evidence gathered amongst adolescents with mental and physical health problems in school settings [37].

In the last decade, some relevant qualitative studies have been carried out to explore the effects of mindfulness programs on young people in schools. For instance, Broderick and Metz [39] gathered feedback from adolescents at an all-girls school who participated in the pilot trial of the *Learning to BREATHE* program. The adolescents experienced a high degree of satisfaction by learning the stress coping strategies used to let go of difficult thoughts and feelings. Similarly, Monshat et al. [40] tried to gain a detailed understanding of what a group of 11 young people between the ages of 16 and 24 had learned about the concepts and practice of mindfulness in a six-week program. The authors identified three stages in the participants’ learning: firstly, they recounted their experiences prior to the practice of mindfulness, which were related to certain negative emotional reactions (e.g., loss of control, exaggerated reactions, and isolation); secondly, issues related to relaxing and conscious emotional control emerged (e.g., stress management, a sense of inner calm, and a greater degree of conscious control); thirdly, key themes were found relevant for the participants’ personal life (e.g., mental clarity, confidence, competence, calm, balance, and control).

Recently, Dariotis et al. [41] presented the results of a mindfulness and yoga-based intervention aimed at fifth and sixth grade school groups including 22 pupils and nine teachers. The analysis of data obtained from focus groups revealed four predominant themes: (1) learners were able to recall breathing exercises and postures more easily and frequently than the discussion topics included in each session; (2) learners identified the connections between the benefits of practices and physical discomfort, recognizing which techniques were most appropriate for personal use or for teaching others; (3) learners reported a sharper emotional assessment of themselves and others, which allowed them to reduce negative emotions and promote positive social interaction strategies that were applied both inside and outside the classroom; and (4) both students and teachers reported realistic and optimistic expectations about the impacts of the intervention, both in the short and long term. Using staged focus groups after a 6-week Paws b program in a state primary school in Wales, Hutchinson et al. [42] interviewed fifteen 10–11-year-old children aiming to understand how mindfulness was helpful in managing the challenges in their daily lives. Emotion regulation was found to be the principal theme of the data analysis, with four themes being identified: (1) elements of the emotion regulation process –including feeling good, present moment focus, stepping back, and the possibility of intentional action, (2) the fact that dysregulation prompted participants to apply mindfulness, (3) challenges and strategies, and (4) the conditions that support or hinder mindfulness use.

The results of these studies, apart from providing an account of the benefits and knowledge acquired by the participants, revealed the ways in which they were able to apply it to various aspects of their personal and social experience.

Even though these studies have provided insights into the subjective process of change that is experienced by adolescents, including some of its most valued aspects (e.g., the applicability of mindfulness tools to daily life), further research is required in order to explore the teaching and learning process involved, as this would provide a deeper understanding of the mechanisms of action of mindfulness practices [42,43]. Specifically, one of the key aspects of mindfulness-based interventions is their pedagogical structure: the specific combination of practices, concepts, and didactic methods used to deliver the intervention [44]. In fact, a meta-analysis by Carsley et al. [15] showed that individual differences and program characteristics can impact the acceptability and effectiveness of mindfulness training. These characteristics included: (a) age of application (better during late adolescence, (b) a combination of different mindfulness activities, and (c) differences in specific outcomes when delivered by an outside instructor compared to trained educators/teachers.

However, these variables have not been sufficiently studied from the perspective of the participants themselves, which is necessary in order to understand the relevance of these aspects for the acquisition of mindfulness skills. It is therefore essential to collect information that considers the specific nature of this age group [16] from a perspective that is sensitive to the social and cultural context in which mindfulness-based interventions take place. This initial step should make it possible to establish cross-cultural comparisons of mindfulness-based interventions within school settings, which would be especially relevant considering that there is virtually no literature on the effect of mindfulness on the subjective experience of adolescents in Latin America.

The aim of this study was to understand the subjective experience of a group of adolescents taking part in a mindfulness-based intervention called “.b curriculum” (pronounced dot-be and explained below), developed to be implemented in school contexts. Specifically, we focus on the following factors: benefits and difficulties that they may have experienced; the atmosphere during the sessions; the relationship established with the other participants and the teacher, among other factors; the way to achieve the perceived benefits; and recommendations for improving the intervention. This objective was guided by the following research questions: (1) How is the pedagogy used in the mindfulness-based intervention perceived by the participating adolescents?; (2) What are the main effects perceived by the adolescents after participating in the mindfulness-based intervention?; and (3) What are the main mechanisms of action associated with the effects reported by the participants?

## 2. Methods

The present study used a qualitative methodology in order to understand the subjective experience of a group of adolescents who voluntarily took part in a mindfulness-based intervention developed to be implemented in school contexts. The research design was exploratory-descriptive and discovery oriented from a hermeneutic-phenomenological perspective [45,46]. Phenomenology is an approach in which the perspective of the participants in a specific phenomenon is considered in all its richness and particularity since it remains as close to the lived experience as possible. The hermeneutic approach, additionally, starts by acknowledging that each perspective is one possible interpretation that must in turn be interpreted by researchers both respectfully and creatively [36]. In this sense, as Fine [47] states, this approach does not provide direct access to the perspectives of others, insofar as researchers inevitably edit and organise participants’ data based on their particular framework of reference. Thus, this methodology is aimed at describing adolescents’ subjective experience and identifying meaningful themes grounded in that experience with the purpose of understanding the three aforementioned aspects of this particular mindfulness-based intervention.

The data resulting from the production stage were analysed using Thematic Analysis [48,49] by inductive categorization [50,51], which is characterized by the construction of emerging categories (inferred from the content), not established a priori using theoretical references. The end result of a thematic analysis is the description of a saturated set of themes, regardless of the frequency of the emergent themes [52,53].

### 2.1. Participants

This study is part of a larger research project aimed at evaluating the feasibility and effectiveness of the application of mindfulness in the Chilean school system. A convenience sampling strategy was used for selecting a sample which met the following inclusion criteria: (a) both genders; (b) 12 to 14 years and 11 months of age; (c) students in their third and fourth year of secondary school; and (d) from both subsidised and unsubsidised schools (with and without government funding, respectively). The parents were asked to sign an Informed Consent form, while the adolescents were asked to sign an Informed Assent form. With these safeguards in place, 47 students started the mindfulness training program, but six did not complete it because the workshop did not meet their expectations. Of the 41 participants who finished the intervention, 22 (54%) agreed to be interviewed, but two were not present the day of the interview. Thus, the total sample of this study consisted of 20 students (11 female and nine male) from two schools in the city of Santiago, Chile (11 from a private unsubsidised school and nine from a private subsidised school).

### 2.2. Characteristics of the Intervention

The mindfulness-based intervention was implemented using a workshop format of eight weekly sessions lasting 45 minutes each (see Table 1). All sessions were conducted within normal school hours and in the students’ usual classrooms. During the first session, there was an introductory talk about mindfulness and its benefits. The main reasons for students not to participate, despite being invited, were a lack of motivation or failure to obtain parental consent. These students performed an alternative activity in the school library, under the guidance of their trainee teacher. The “.b curriculum” used in this study is part of a larger program developed in the UK, known as Mindfulness in Schools Project [54]. This course was specifically designed by teachers to teach secular mindfulness to adolescents between 11 and 18 years old in school contexts. The word “secular” is used to mean implementations of mindfulness based on a scientific framework, generally informed by psychology and neuroscience, not by spiritual traditions such as Buddhism [55,56].

The “.b curriculum” contributes to skill development through exercises and elements common to other MBIs that have been adapted to this population. Particularly, in the .b program (following the Mindfulness-based Stress Reduction Program [44] approach), each workshop session was developed around a central theme, making use of specific visual learning aids (slides). Segments of films were included in some sessions to illustrate the underlying theoretical concepts, while metaphors were frequently used to the same end. In each session, both formal and informal mindfulness exercises are taught. The formal practices are time-limited (approximately 10 min.) and are used to train awareness of bodily sensations, emotions, and thoughts. (e.g., body scan, mindful movement, sitting meditation). Informal practices include tooth brushing, mindful eating, and dish-washing, among other activities, which help cultivating present moment awareness in daily life. Moreover, each participant was given a notebook containing a summary of each session and the exercises to be done at home. Audio recordings containing key meditative practices were also provided. All sessions were conducted by a psychologist certified in the use of the “.b curriculum”.

### 2.3. Data Collection

Data were produced using semi-structured in-depth interviews conducted one week after the intervention, using guidelines with open-ended questions developed to focus the conversation on the contents and activities of the workshop. This should make it possible to explore the participants’ perceptions about the workshop experience in the following areas: (a) benefits and difficulties that they may have experienced (e.g., Did you find any benefits to be involved in the workshop? Did you find any difficulties during the workshop?); (b) the atmosphere during the sessions (e.g., How was the atmosphere during the sessions?); (c) the relationship established with the other participants and the teacher (e.g., How was the relationship with your peers and the teacher (instructor) of the workshop?), among other factors; and d) recommendations for improving the intervention (e.g., Do you have any recommendation to improve the workshop?). The participants were asked about the areas or situations in which they reported benefits and how they achieved those benefits. For example, if a participant reported having obtained concentration-related benefits during tests at school, the interviewers sought to obtain as much detail as possible about this issue. Finally, the participants were asked to suggest how the workshop’s pedagogical approach could be improved for future interventions.

All interviews were conducted by two psychologists trained in the use of the guidelines and with experience in research interviews (with no involvement by the .b instructor). The interviews lasted approximately 20 minutes each and were audio-recorded for later transcription, in order to initiate the coding process.

### 2.4. Data Analysis

The data resulting from the collection stage were analysed following a procedure informed by thematic analysis [39], which is a broad and flexible “[…] method for identifying, analysing and reporting patterns (themes) within data” [49,53]. Thematic analysis is compatible with many different methodological approaches, such as the hermeneutic-phenomenological perspective based on the interpretative qualitative paradigm used in this study. To obtain meaningful knowledge, this approach combines a precise description of the participating adolescents’ perception with a rigorous interpretation of these discourses in order to construct relevant themes and sub-themes. Following Braun et al. [57], the analysis was carried out in six phases: (1) become familiar with the data and identify words of potential interest; (2) generate initial codes for each speaking turn (unit of analysis), in order to identify important characteristics of the data; (3) look for broader patterns of meaning by examining and comparing previously generated codes; (4) review the themes and apply them to the data set to determine if they are compelling; (5) decide on the final name of each theme; and (6) integrate the analytical narrative with the existing literature, supported by the use of illustrative vignettes. This analysis process involved two members of the research team (the mindfulness workshop instructor and one of the interviewers), whose results were subsequently triangulated and agreed upon with a third external coder with vast experience in qualitative research. The external coder acted as an auditor to check the work of the primary team of raters and minimize the effects of groupthink in the primary team [58]. For instance, when a disagreement was detected, the meaning of the narrative was discussed by the primary team first, selecting only the categories in which there was agreement. Consensual categories were triangulated again with the external coder. Finally, only those categories with total consensus among the three researchers were kept. Thus, the scientific accuracy of our qualitative analyses was based on intersubjective agreement, a relational process aimed at reaching a comprehensive understanding of the phenomena examined [58].

### 2.5. Ethical Approval

The project “Feasibility and effectiveness of the use of mindfulness in the Chilean school context” (n° 82130055), to which this study belongs, was approved by the Ethics Committee of the Faculty of Psychology of the Pontificia Universidad Católica de Chile.

## 3. Results

The resulting key areas, themes, and subthemes were grouped according to the research questions as follows: (1) meanings associated with the pedagogical approach used during the workshop; (2) participants’ views on the main effects of the intervention; and (3) main mechanisms of action associated with the process of practising mindfulness. Some representative quotations were included to illustrate some of these themes (see Table 2). Additionally, the adolescents’ views on how the workshop could be improved for future interventions were presented as part of research question 1.

### 3.1. Key Area 1: Pedagogical Approach of the Intervention

It was observed that, for most participants, what was taught during the workshop (e.g., attention, difficult emotions) was as relevant as the way in which this content was conveyed. Thus, participants highlighted the fact that different visual materials used during the workshop (e.g., slides, film segments) were useful in order to capture their attention throughout the entire learning process. The metaphorical contents (e.g., mind as a pet, sports training) were evaluated positively by the participants, since they helped them to recognise mindfulness concepts in everyday situations or in animal behaviour. The following vignettes allow us to illustrate the above: “*we should continue learning how to work on this* [mindfulness skills] *more, and also the activities we did were fun*” (S6, female), “*The slides they showed us were fun… because they weren’t literal*” (S5, female), and “*The example of a torch, I think that helped a lot for everyone to understand how it* [attention] *worked…*” (S6, female).

The structure of the workshop was another of the aspects perceived positively by the participants, including the planning and design of the program (e.g., “*I liked the way they planned it*”, S5, female; “*Very effective, very good, very simple*”, S3, male). A well-defined structure allowed participants to feel that they were in a learning environment that was conducive to psychological well-being, beneficial to them, and potentially useful to adults (e.g., their parents). Specifically, the workshop sessions were subjectively experienced by the participants as situations characterized by trust and security, allowing them to freely express what they thought or felt. The workshop in its entirety was valued as an opportunity for growth (e.g., “*we were able to learn a lot with this workshop in every session that we had*”, S6, female) and self-improvement (e.g., “*From my point of view, the programme is well done because it goes through stages… it’s like you are steadily learning the techniques in order to improve*”, S5, female).

Participants reported that, as the workshop progressed, they experienced a gradual increase in mindfulness capacity. While they initially found some activities more difficult than others, the participants were able to perform them little by little. In this regard, one of the participants pointed out: “*at the start, in the first session, I couldn’t actually do any of the things I was asked to do, I couldn’t control myself, but by the last session I was really relaxed and I did everything well*” (S5, female).

Regarding the practical activities carried out during the workshop, these were perceived by the participants as useful and significant. With respect to this, one participant said that “*activities like lying on the floor, concentrating on my body, on what my body is doing, were one of the main factors that helped me*” (S6, female), while another participant stated that the workshop was “*like a life experience, because everything that is covered is useful…*” (S6, female). Interestingly, the participants did not regularly perform formal mindfulness practices outside the workshop (e.g., body scanning technique); however, they were able to conduct informal mindfulness practices in their daily life (e.g., walking down the street, washing dishes), which were also more widely valued (“*I learned to realise things I had never realised. For example, when I was walking, I didn’t notice the places that I passed, nor did I give myself the time to see well*” (S6, female). Another important aspect mentioned by the participants was the degree of motivation needed to achieve the results expected from practical activities. Regarding this, one participant noted that “*only those who really wanted to achieve something with this stayed in the program*” (S4, male).

Another positive aspect highlighted by the participants was the instructor’s expertise in leading the group. One of the manifestations of this expertise was the ability to lead group mindfulness activities (e.g., demonstrating the difference between reacting and responding to distractions in the group) or the ability to show participants how to transmit their own experiences (e.g., when they shared how they had reacted in certain situations). Participants also stressed the importance of the instructor’s attitude in establishing a positive relationship between him and the group (e.g., showing patience, acceptance, and kindness). Regarding this dimension, one participant said: “*It was good, he is really friendly, and talking with him is not like a debate, it is like speaking freely, and nothing we say is wrong*” (S3, male). Likewise, other two participants noted the following about the instructor: “*Yes, he was kind and a good psychologist. He was calm*” (S11, female) and “*…with him, it was easy to say things because we were in confidence*” (S16, male).

Moreover, the adolescents’ views on how the workshop could be improved for future interventions included aspects of the structure of the intervention (e.g., increasing the frequency and number of classes) and conveyed their positive attitude towards this kind of intervention (e.g., ensuring the continuity of these initiatives). Other suggestions referred to aspects of the material, specifically the improvement of worksheets, school environment variables (e.g., using less noisy rooms), and characteristics of the activities presented in several lessons of the .b curriculum (i.e., more activities and less relaxation, more chocolates, and more options and freedom for some positions). Interestingly, some respondents said that they would not change the intervention in any way.

### 3.2. Key Area 2: Perceived Effects

One of the cognitive effects that participants perceived most often was that practising mindfulness allowed them to increase attention and concentration, for example, when teachers presented new content in class (e.g., “*I am more aware of the things I do*”, “*I pay more attention to the lessons than before*”, “*I have been more aware of the things they teach me.... before I got distracted a lot*”; S6, female). One of the participants said the following about school tests:

“During tests, I used to think about other things, I didn’t concentrate and made stupid mistakes, and now it’s like I’m concentrating more, and instead of telling myself not to think about something, I let it flow and start thinking about what I have to do, and I’m doing better” (S6, female).

Improvements in problem solving were also perceived by the participants, who felt that they had more tools at their disposal when it came to finding solutions to their difficulties. For example, they learned to prioritize that which is more important than secondary concerns (e.g., “*I started to sort my ideas from the most important to the least important, that’s what needs to be resolved now*”; S7, female). In addition, some participants were able to re-signify experiences that had previously caused them difficulties or that they had experienced as negative. For example, one participant reported that he was concerned about what people thought of him before (“*people’s comments used to affect me a lot, and now I don’t care, that has helped me a lot*” (S3, male).

Effects were also perceived at the emotional level, where a greater sense of calm and well-being stood out. Specifically, participants reported experiencing a sense of growth (“*I feel that I needed to mature, and with this program I was able to mature more and grow in many ways*”; S3, male), greater self-confidence (“*I now have more confidence in myself to be able to do things*”; S2, male; “*It gives me a sense of security, the security that I can do it*”; S2, male), and more happiness and gratitude (“*I feel happier with myself, I feel more grateful with what I have*”; S3, male).

Regarding somatic effects, the greatest benefit perceived by the participants was the feeling of body relaxation (“*one feels more relaxed, lighter*”; S3, male), which in turn had a mental effect (“*everything was relaxing and my mind was no longer entangled*”; S3, male). Some participants also reported an improvement in sleep quality, as it took them less time than before to fall asleep. The participants expressed the following regarding the benefits of body exploration exercises: “*I started integrating it* [mindfulness practice], *and now I fall asleep almost immediately*” (S6, female), “*It allowed me to feel sleepy, and my idea was to make my mind go blank, so to speak, to help me sleep, because it’s like I’m always thinking and thinking, and I can’t fall asleep*” (S3, male).

### 3.3. Key Area 3: Mechanisms of Action

The third research question concerned the exploration of mechanisms of action, especially the regulatory strategies related to the perceived effects of mindfulness practice. In this context, body awareness facilitates an attentional shift towards present moment bodily experiences (particularly conscious breathing and body relaxation), which emerge as new attentional focuses that make it possible for difficult experiences to be processed (“*with my breathing, closing my eyes, releasing my muscles, that’s where I felt more relaxed, I felt different than before*”; S3, male). Participants stated that, mainly through the joint or independent application of these two somatic dimensions (i.e., conscious breathing and body relaxation), it was possible to achieve the effects mentioned above (e.g., a greater sense of calm): “*I tend to get very angry and argue, so when I’m arguing I calm down, breathe, close my eyes, and that has helped me interact better with others*”; S6, female).

**Figure 1:**
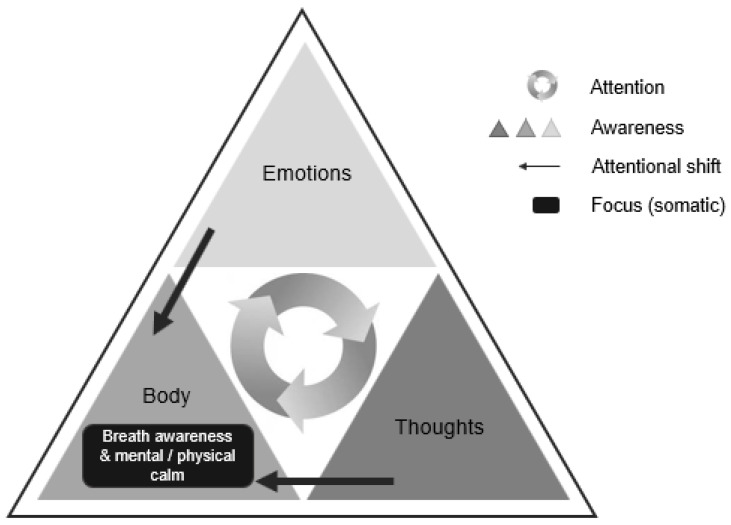
Model of regulatory strategies implemented by adolescents.

Figure 1 shows how the participants explained their learning achievements. This largely top-down process of cognition and emotion for experiencing one’s own body illustrates the regulatory strategies that were learned and used by participants during the workshop, reflecting new ways of handling difficult situations. The circle represents the capacity of the reactive states of awareness within different cognitive, emotional, and somatic levels (e.g., rumination, irritation, tension), while the triangle represents the process through which participants redirect their attention from the cognitive and emotional domains to bodily sensations and breathing as a more direct way of becoming aware of those mental processes and regulating experience (e.g., “*when I read I think of something else, and then I have to read it all over again, but I count my breaths and my heartbeats and all those things* [exercises], *and I concentrate more*”; S6, female).

## 4. Discussion

**Figure 2:**
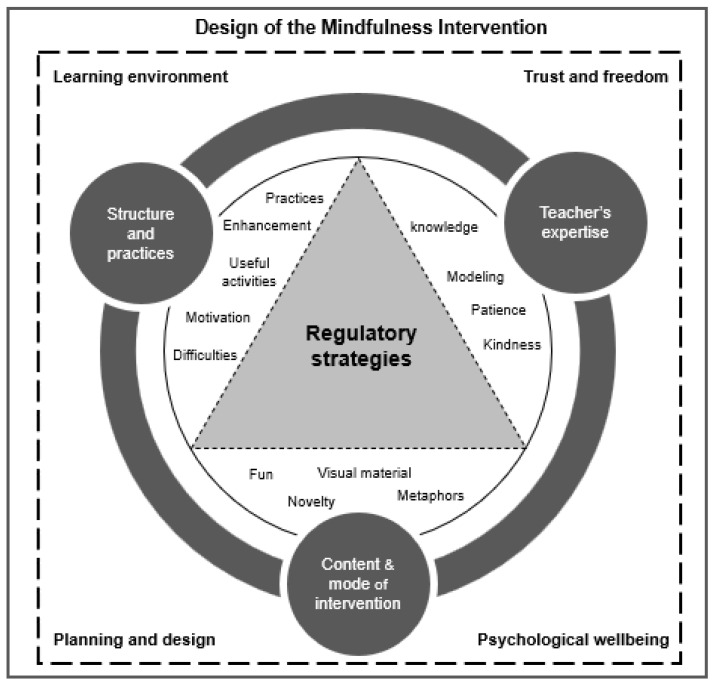
Model of mindfulness-based intervention for adolescents.

This study explored the perceptions of a group of adolescents from two schools in Santiago, Chile, who participated in a mindfulness-based intervention called dot-b. The model presented in Figure 2 considers four aspects of the pedagogical approach used during the intervention (contents/mode, structure, practices/activities, and instructor characteristics), which are configured to explain the relational-pedagogical framework on which the intervention is based.

This relational framework made it possible to provide adolescents with a safe environment, based on trust and acceptance, which allowed them to gradually develop multiple regulatory strategies that they themselves described as the mechanisms of action that made it possible to achieve the effects perceived at the cognitive, emotional, and somatic levels. These effects reinforced not only the intervention itself, but also the conscious practices that they applied in their daily lives. In other words, the participants mainly connected the perceived benefits with the type of pedagogy used during the workshop.

Insofar as the pedagogical approach used to implement mindfulness-based interventions in school contexts –one of the most important elements to ensure effectiveness– has received little empirical attention, it is necessary to understand the process of personal development experienced by adolescents during the intervention [16,18,38,40,41,42]. In fact, our findings resonate with the meta-analysis conducted by Carsley [15], showing that differences between the characteristics of mindfulness programmes have an impact on participants’ responsiveness and training effectiveness. Even though the scope of our study was not to measure effectivity, our qualitative results reassure the use of a pre-designed mindfulness program (.b) when delivered by an experienced facilitator. In our case, the instructor’s mindfulness practice had a relevant impact on the mindfulness intervention. Thus, we agree with Carsley’s idea that it is necessary for existing mindfulness programs to rely on the facilitator’s ability and familiarity with mindfulness.

One of the central aspects of the learning process during the workshop was the applicability of the mindfulness tools presented to the participants’ daily life. Results were very similar to those found with young people in the USA after interventions of this type [41]. This observation highlights the importance of placing learning outcomes in contexts that are as natural as possible in order to facilitate the intention to be here and now. This involves incorporating motivational elements into the design of the intervention, for example, demonstrating the advantages of applying mindfulness tools in daily living activities and emphasizing their benefits at the academic level, in stress management, in sports or musical performance, and even in establishing better interpersonal relationships with peers [41]. This would also create clear expectations about the acquisition of mindfulness tools as a process that occurs gradually, requiring practice and motivation from participants [40]. Therefore, it is possible to suggest that informal practices are at least as important as formal practices regarding the benefits perceived by adolescents within the school context, which are mainly related to emotion self-regulation [59]. This means that, besides the content of the program, the form of implementation and community support are crucial factors for its success. Thus, following Hutchinson et al. [42], attention must be paid to informal practices beyond the program’s sessions; to do so, researchers must find useful ways to introduce them into the entire school community and implement them during the classroom activities of every subject. Being cross-curricular, these practices need to be easy to implement and transfer temporally and spatially –to other schools and to everyday situations of pupils’ life. This feedback is valuable when it comes to identifying and working on possible sources of frustration experienced by participants in situations where they perceive themselves as “unable” to meet their expectations regarding the practical exercises.

It was observed that the key element that links the different elements taught during the workshop (both in content and form) is the figure of the instructor. The relevance of the instructor’s personal mindfulness practice has always been one of the focuses of interventions based on mindfulness [44,60], as the cultivation of a mindful teacher presence within the educational setting represents a permanent necessity and challenge [61,62] in order to offer concrete examples of what is being taught during the workshop [63]. The teacher’s self-disclosure appears to generate greater trust and a more horizontal relationship with the participants, who can explicitly and implicitly recognize the teacher’s humanity, rather than a vertical relationship in which the teacher is perceived as a figure of complete authority.

Regarding the effects of the intervention perceived by the participants, they were in line with those found in qualitative and quantitative studies, in which an increase in cognitive and emotional capacities has been reported [15,16,18,40,41]. There is evidence that an increase in participants’ cognitive functions (e.g., level of attention) is associated with improved academic performance and overall performance in school activities [19,20,21,22]. Similarly, mindfulness training has enabled participants to improve their problem-solving skills in a variety of academic and relational contexts (e.g., with peers and friends), as they learned new strategies for coping with the difficulties that they face in their daily lives. Thus, when dealing with experiences that may cause distress, the participants narrate how mindfulness helps them move from a state of aversion to a state of acceptance in their inner experience [41].

Among the emotional benefits experienced by the participants, they reported a greater sense of well-being, calm, and, above all, a sense of personal growth based on better self-confidence, happiness, and gratitude. These benefits are part of the development of social-emotional competencies [21,25,27,64,65]. Furthermore, at the somatic level, adolescents reported positive effects associated with sleep quality and body relaxation. Since sleep quality issues are common among children and adolescents, this valuable outcome highlights the benefits of these types of interventions for the development of several essential psychophysiological functions during this stage of development [66,67].

The *mechanisms of action* behind the effects perceived as a result of practising mindfulness were also explored. In this age group, the body plays a fundamental role in the acquisition of mindfulness skills. The body can become a resource for adolescents to identify and interpret their inner states and deal with them in a healthy way. Thus, body relaxation and conscious breathing are used to address the process of change from states characterized by deregulation to states of greater calm and integration [68]. Current findings provide further evidence highlighting the role of the body, thus complementing results from Hutchinson et al. [42] regarding processes involved in emotion regulation in children. More specifically, access to the somatic experience, as reported by the participants in this study, suggests that present moment self-awareness is largely based on the body. Thus, body awareness plays a fundamental role in the recognition and regulation of emotional and cognitive processes [14]. In other words, adolescents’ bodies could work as the main attentional anchor enabling them to become aware of their mental and relational states at a more direct *level* than adults. Given the association between negative psychological and/or emotional states and somatic symptoms during adolescence [69], it seems essential to continue exploring the role that mindfulness might play in how adolescents relate to their own bodies. In fact, in adults, greater body awareness plays a relevant role in the emotional regulation process, since recognition of one’s affective states is a prerequisite for self-regulation (e.g., [70,71]). This argument is in line with studies that argue that increased interpersonal awareness is related to improved psychological well-being (e.g., [72]).

One of the limitations of the present study was its cross-sectional design, which made it impossible to verify whether the participants’ perceptions changed over time and, specifically, prevented us from determining whether the perceived benefits at the end of the intervention were maintained as a consequence of additional practice. Future studies should adopt a prospective design and include control groups to explore changes over time and the specificity of the dot b mindfulness-based intervention. Additionally, future studies could focus on identifying mechanisms of action or determining which training delivery approaches are the most effective. Concerning mechanisms of action, the use of biomarkers of autonomic nervous system (ANS) state (i.e., parasympathetic vs sympathetic) could provide a comprehensive picture for the assessment of mindfulness training efficacy, enabling researchers to match ANS functioning with the reported subjective experience. Moreover, quantitative studies have shown that those adolescents who practice more outside the workshop end up benefiting more than those who discontinue the practice [73]. A challenge for future research is to identify factors related to the maintenance or abandonment of the practice of mindfulness over time, in order to be able to consider these aspects when designing and implementing comprehensive care programs in schools. Another limitation was the absence of formal interviews with teachers and/or family members, who could have provided more information about the perceived effects of the workshop on their students or children, thus complementing the adolescents’ report. It should be noted that all students who took part in this intervention did so voluntarily, but only some of them agreed to be interviewed. Therefore, interviewing the most motivated adolescents, or those who felt that they benefited most from the intervention, is likely to have produced a bias in the data collected in this study. This appears to be a common limitation in this type of study, since it has been observed in other recent qualitative studies on adolescents and mindfulness [40,41]. Additionally, the sessions were not externally evaluated to determine fidelity to protocol, although they were conducted by a certified instructor. We did not measure the participants’ subjective experience after each session. Relevantly, it is important to be cautious with the potential bias derived from the fact that the instructor was involved in the data analysis process. Being aware of this risk, we used the instructor’s perspective as a relevant element for grasping the meaning of the adolescents’ voice. The validity of this process was permanently ensured through triangulation. In this regard, we intend to reduce the distance between the researcher and the researched through an accurate interpretative process [74]. Furthermore, future studies should address the relevance of home practice in adolescents, for instance, by weighting the role of informal and formal assigned practices. In this vein, further research is needed to explore how to include students who need more flexibility of the curriculum, for instance “more options and freedom for some positions” S8; male.

Finally, it should be noted that the results obtained coincide with those of studies carried out in other cultural and socioeconomic contexts (e.g., USA, Europe). This reinforces a basic conception of mindfulness as a set of universally attainable skills that constitute an important resource for prevention and promotion in the sphere of adolescent physical and mental health [75]. Additionally, these results may contribute to the scientific understanding of the implementation process of mindfulness within schools. Specifically, they are relevant because the .b curriculum was developed in a different cultural background and its implementation should consider the voice of Chilean adolescents. A more explicit examination of the cultural specificities of this kind of studies remain a relevant challenge for future work in this field.

## 5. Conclusions

In summary, these results support the view that pedagogy is a very relevant consideration in the implementation, development, and efficacy of mindfulness-based interventions within school contexts. We propose that the inclusion of structure, contents, process/mindful practices, and teachers’ expertise provides the pedagogical-relational framework required for students to successfully develop mindfulness skills, which enables them to experience their cognitive, emotional, and somatic effects. According to our results these effects are linked to self-regulation strategies, based on paying attention to one’s somatic experience with kindness and curiosity, which works as an attentional anchor. It is hoped that these results will contribute to the spread of mindfulness research in adolescents in Latin America, thus facilitating cross-cultural and international comparisons.

## Figures and Tables

**Table 1:** Themes of each .b session.

Orientation	An Introduction to Mindfulness
Lesson One	Playing Attention
Lesson Two	Taming the Animal Mind
Lesson Three	Recognising Worry
Lesson Four	Being Here Now
Lesson Five	Moving Mindfully
Lesson Six	Stepping Back
Lesson Seven	Befriending the Difficult
Lesson Eight	Pulling it all Together

**Table 2:** Key areas, themes, and sub-themes.

Key Areas	Themes	Sub-Themes
I. Pedagogical approach of the intervention	Content and mode of intervention	Visual material Metaphors
	Structure of the intervention	Planning and design Learning environment Gradual process
	Practical activities	Useful activities Pre-eminence of informal practices Motivation as requirement
	Teacher’s expertise	Group management Mindfulness skills modelling Attitudes of acceptance and kindness
II. Perceived effects	Cognitive effects	Attention and concentration Problem-solving Resignification of experience
	Emotional effects	Sense of growth and self-confidence Happiness and gratitude
	Somatic effects	Body relaxation Quality of sleep
III. Mechanisms of action	Body awareness and attentional shift	Breath awareness Body relaxation Mental and physical calm
